# Ameliorating Effects of *TRIM67* against Intestinal Inflammation and Barrier Dysfunction Induced by High Fat Diet in Obese Mice

**DOI:** 10.3390/ijms23147650

**Published:** 2022-07-11

**Authors:** Qihui Luo, Asad Jahangir, Junbo He, Chao Huang, Yu Xia, Lanlan Jia, Xiaoli Wei, Ting Pan, Yanni Du, Bin Mu, Huan Gong, Wentao Liu, Saif Ur-Rehman, Kangcheng Pan, Zhengli Chen

**Affiliations:** 1 Laboratory of Experimental Animal Disease Model, College of Veterinary Medicine, Sichuan Agricultural University, Chengdu 611130, China; lqhbiology@163.com (Q.L.); 2019603003@stu.sicau.edu.cn (A.J.); junbohe19@163.com (J.H.); xiayu113bvs@163.com (Y.X.); jialanlan2015@163.com (L.J.); wei_xl323@163.com (X.W.); panting555666@163.com (T.P.); DuYanni0026@163.com (Y.D.); tiaoms7@163.com (B.M.); gh31071@163.com (H.G.); liuwt1986@126.com (W.L.); 2 Key Laboratory of Animal Disease and Human Health of Sichuan Province, College of Veterinary Medicine, Sichuan Agricultural University, Chengdu 611130, China; pankangcheng@sicau.edu.cn; 3 Department of Parasitology and Microbiology, FV&AS, PMAS Arid Agriculture University, Rawalpindi 46300, Pakistan; drsaif578@uaar.edu.pk

**Keywords:** high fat diet, *TRIM67*, knockout, intestine, inflammation

## Abstract

Tripartite Motif 67 (TRIM67) is an important member of TRIM family proteins, which participates in different cellular processes including immune response, proliferation, differentiation, carcinogenesis, and apoptosis. In recent years, a high fat diet (HFD) has remained one of the main causes of different metabolic diseases and increases in intestinal permeability as well as inducing intestinal inflammation. The current study investigated the protective effects of *TRIM67* in the ileum and colon of obese mice. 4-week-old wild-type (WT) C57BL/6N mice and *TRIM67* knockout (KO) C57BL/6N mice were selected and randomly divided into four sub-groups, which were fed with control diet (CTR) or HFD for 14 weeks. Samples were collected at the age of 18 weeks for analysis. To construct an in vitro obesity model, over-expressed IPEC-J2 cells (porcine intestinal cells) with *Myc-TRIM67* were stimulated with palmitic acid (PA), and its effects on the expression level of *TRM67*, inflammatory cytokines, and barrier function were evaluated. The KO mice showed pathological lesions in the ileum and colon and this effect was more obvious in KO mice fed with HFD. In addition, KO mice fed with a HFD or CTR diet had increased intestinal inflammation, intestinal permeability, and oxidative stress compared to that WT mice fed with these diets, respectively. Moreover, IPEC-J2 cells were transfected with TRIM67 plasmid to perform the same experiments after stimulation with PA, and the results were found consistent with the in vivo evaluations. Taken together, our study proved for the first time that HFD and *TRIM67* KO mice have synergistic damaging effects on the intestine, while *TRIM67* plays an important protective role in HFD-induced intestinal damage.

## 1. Introduction

Obesity is a major evolving public health problem in developed countries. The etiology of obesity can be defined as the imbalance between energy consumed in food and energy expended. The extra energy is stored in fat cells [1], and a high fat diet (HFD) is a primary contributing factor for obesity [2]. The intestinal tract not only absorbs essential nutrients from food, but also protects the host from ingested toxins and microbes. It is a multi-faceted barrier, composed of a mucus layer, a constantly renewing epithelial boundary and tight junctions, which work to protect intestinal health [3]. Dietary fat disrupts tight junctions in the intestinal epithelium and leads to increased intestinal permeability [3,4]. Previous studies have reported that HFD promotes inflammation in the gastrointestinal tract which is directly linked with HFD-induced obesity [5,6,7,8]. Moreover, high fat diets can lead to atrophy of intestinal villi, oxidative stress, and intestinal barrier disruption [3]. Some studies have demonstrated that HFD directly or indirectly causes intestinal oxidative stress [9] especially in the colon [10].

Tripartite motif (TRIM) family proteins, most of which display E3 ubiquitin ligase activities, play an important role in many biological processes, such as proliferation, differentiation, autophagy, and apoptosis of cancer cells [11]. Recent studies have established that most members of the TRIM family are expressed in reaction to interferons and are associated with innate immunity [12,13]. TRIM family proteins have been classified into 11 groups (from C-I to C-XI) [14], and TRIM67 is a member of TRIM family proteins from C-I [15,16]. The previous studies regarding TRIM family proteins determined that some TRIM family proteins normalize intestinal inflammation, as in *TRIM14* knockout mice that exhibit colitis and colitis-related tumors [17]. A direct contribution of autophagy to intestinal inflammation was previously demonstrated for *TRIM11* [18] and *TRIM14* [17]. TRIM67 is a particular E3 ubiquitin ligase, which is important for target protein ubiquitin-dependent degradation or stabilization. Currently, little is known about *TRIM67*, although a previous study has proved that *TRIM67* has been silenced in colorectal cancer [19], and is an important member of tumor suppressive TRIMs [20]. *TRIM67* is essential for proper brain development and behavior [21]. Increasing evidence shows the vital role of TRIM proteins in the regulation of the NF-κB signaling pathway, and a recent study showed that *TRIM67* is a negative regulator of TNFα-triggered NF-κB activation, while deletion of *TRIM67* enhances inflammatory cytokines [22].

The inflammatory effects of a HFD and ameliorating effects of *TRIM67* on inflammation have been described by previous studies. However, to the best of our knowledge, the ameliorating effects of *TRIM67* on intestinal inflammation due to HFD feeding have not been reported yet. Therefore, the current study aimed to elucidate the protective effects of *TRIM67* in amelioration of the intestinal inflammation and barrier dysfunction induced by HFD in obese mice.

## 2. Results

### 2.1. High Fat Diet (HFD) Suppresses the Expression of TRIM67

**Figure 1:**
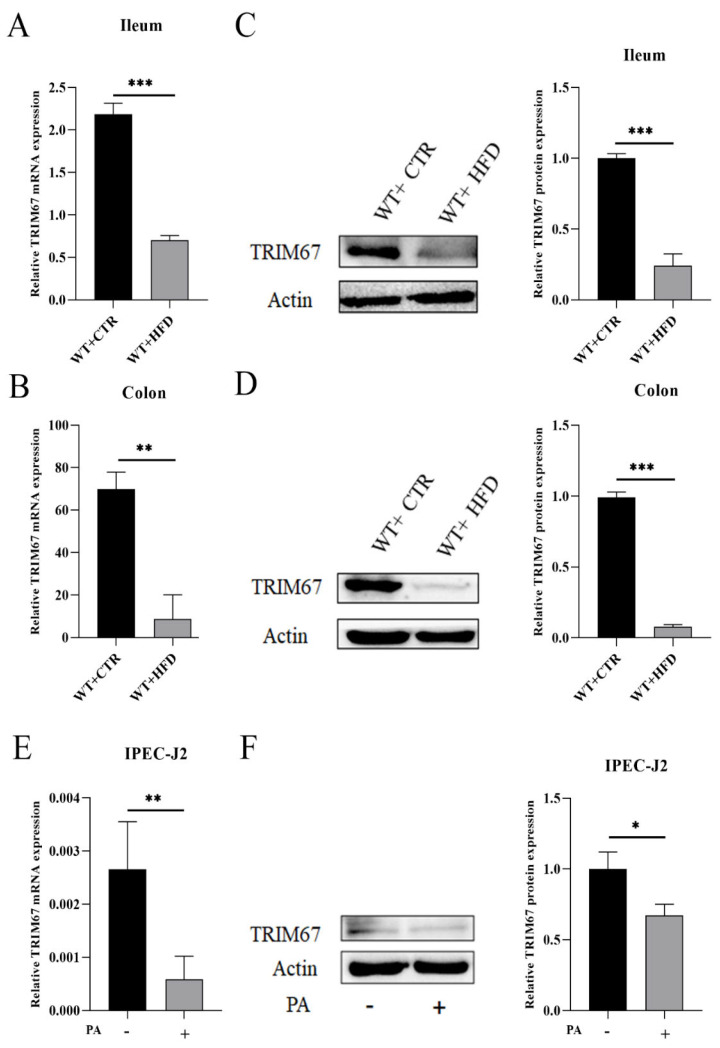
Effect of HFD on the expression of *TRIM67* in WT mice. (**A**,**B**) Relative mRNA expression of *TRIM67* in ileum and colon (*n* = 4). (**C**,**D**) WB and quantification of TRIM67 in ileum and colon of WT CTR and WT HFD groups (*n* = 4). (**E**) IPEC-J2 cells were supplemented with PA to develop an obesity model in vitro and relative mRNA expression of *TRIM67* in PA supplemented group (*n* = 3). (**F**) WB results and quantification of protein expression of TRIM67 in PA supplemented IPEC-J2 cell lines, “-”: no addition, “+”: addition (*n* = 3). The data are expressed as mean ± SEM. Significance was determined by two-tailed Student’s *t* test (* *p* < 0.05, ** *p* < 0.01, *** *p* < 0.001).

In order to explore the effect of HFD on *TRIM67*, we fed the WT mice with HFD for 14 weeks and developed the overexpressed *TRIM67* IPEC-J2 cells by transfecting the cells with *Myc-TRIM67*. The WT HFD group showed the decreased expression of *TRIM67* in ileum and colon compared to the WT CTR group (Figure 1A,B). Western blotting (WB) results confirmed the significant suppression of TRIM67 protein expression levels in the ileum and colon of the WT HFD group as compared to the WT CTR group (Figure 1C,D).

The IPEC-J2 cell lines were supplemented with PA to develop the obesity model in vitro. Our results showed that the mRNA expression of *TRIM67* was downregulated in PA supplemented IPEC-J2 cell lines (Figure 1E). The relative TRIM67 protein expression also showed a decreased expression of TRIM67 in PA supplemented IPEC-J2 cell lines (Figure 1F). These results suggested that HFD suppressed the expression of *TRIM67*. Therefore, we designed KO mice for our experiment (Supplementary Figures S1 and S2). Taken together, these results indicated that the HFD down-regulated the expression of *TRIM67*.

### 2.2. Deletion of TRIM67 Exacerbates the Pathological Damage of Intestine Induced by High Fat Diet (HFD)

**Figure 2:**
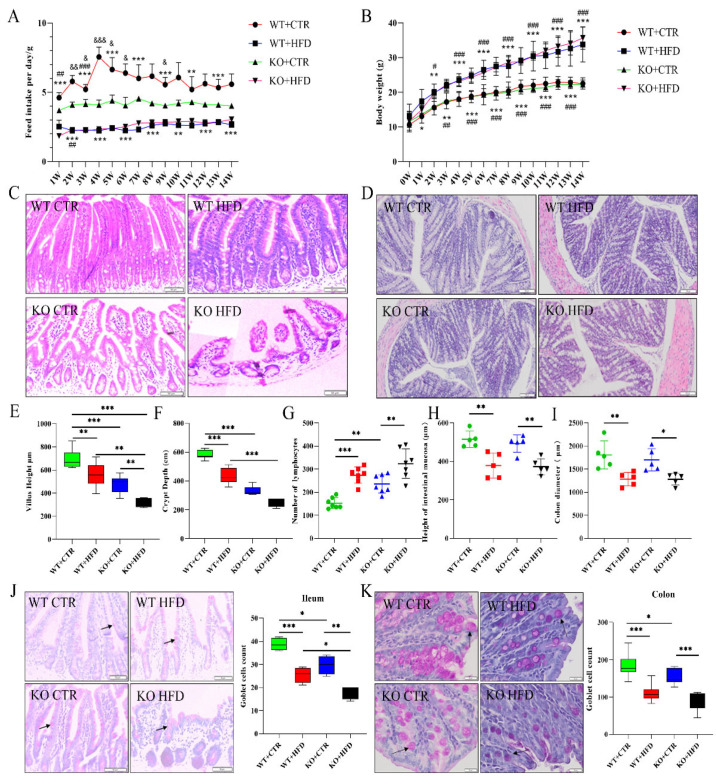
*TRIM67* deletion has no effect on body weight and exacerbates the pathological damage. (**A**) Average weekly feed intake of mice during modeling WT CTR compared to WT HFD (*n* = 8), * *p* < 0.05, ** *p* < 0.01, *** *p* < 0.001; KO CTR compared to KO HFD, ^#^ *p* < 0.05, ^##^ *p* < 0.01, ^###^ *p* < 0.001; WT CTR compared to KO CTR, ^&^ *p* < 0.05, ^&&^ *p* < 0.01, ^&&&^ *p* < 0.001. (**B**) Average weekly weight gain of mice WT CTR compared to WT HFD (*n* = 11), * *p* < 0.05, ** *p* < 0.01, *** *p* < 0.001; KO CTR compared to KO HFD, ^#^ *p* < 0.05, ^##^ *p* < 0.01, ^###^ *p* < 0.001. (**C**) H&E results of ileum indicating the pathological damage in different groups as compared to WT CTR diet group. (**D**) Colon H&E staining results displaying the difference in lymphocyte number, height of intestinal mucosa and colon diameter in different groups. (**E**,**F**) Quantification of villus height and crypt depth in ileum (*n* = 5). (**G**–**I**) The quantification of lymphocyte number, height of intestinal mucosa and diameter in colon (*n* = 5). (**J**,**K**) PAS staining results in ileum and colon and graphical representation of goblet cell count. Black arrows showing the goblet cells in ileum and colon (*n* = 5). The data are expressed as mean ± SEM. Significance was determined by one-way ANOVA (* *p* < 0.05, ** *p* < 0.01, *** *p* < 0.001).

To understand the role of HFD and *TRIM67* in intestinal pathology we used the KO mice and developed the obesity model and fed WT and KO mice with CTR diet as well as HFD for 14 weeks. The feed intake in the WT CTR diet group was significantly higher as compared to all other feed groups while feed intake was almost similar in the HFD groups. Average daily feed intake of WT CTR and KO CTR diet mice was 5.84 g and 4.14 g while it was 2.51 g and 2.61 g in WT HFD and KO HFD groups, respectively. However, there was no significant difference between WT HFD and KO HFD in feed intake (Figure 2A). Moreover, there was no considerable difference in the body weight of KO and WT groups fed with the CTR diet. The weight in HFD-fed groups was same and significantly more than that of CTR diet groups which indicated the successful development of an obesity model (Figure 2B). HFD generally increased the blood glucose levels of mice (both WT and KO groups) relative to CTR diet groups. (Supplementary Figure S3). Under HFD, the blood cholesterol level increased in both WT and KO groups as compared to their respective CTR diet groups. Similarly, we found an increase in high-density lipoprotein (HDL) and low-density lipoprotein (LDL) in HFD groups as compared to the CTR diet group, while no significant difference was observed in blood triglyceride level (TG) (Supplementary Figure S4).

We examined the morphological changes in the intestine of WT and KO groups of mice fed with the CTR diet and HFD. We detected that the intestinal length was significantly shorter in WT and KO groups fed with HFD, and the intestinal length was significantly lower in the KO group than that of the WT group when fed with CTR diet (Supplementary Figure S5). The H&E staining of ileum showed that the intestinal villi became short and blunt in KO mice when fed with CTR diet as compared to WT CTR diet mice, while the HFD feeding to KO mice exacerbated this pathological damage as compared to WT HFD mice (Figure 2C). In colon, the number of lymphocytes, mucosal thickness and colon diameter represent the pathological damage (Figure 2D). On quantification of results, the villus height and crypt depth of ileum declined in the KO CTR diet group compared to the WT CTR diet group and the lowest expression was seen in KO HFD group (Figure 2E,F). Moreover, a greater number of lymphocytes was exhibited by colon in the HFD group relative to the CTR group, and the number was higher in the KO group compared to the WT group (Figure 2G). Both mucosal thickness and colon diameter in the HFD group were significantly lower than those in the CTR group (Figure 2H,I). PAS staining demonstrated that the average goblet cell count in ileum and colon was lower in HFD groups as compared to their relevant CTR diet groups, while the number was lower in KO groups as compared to the CTR group when fed with the CTR diet (Figure 2J,K). This decrease in goblet cell count was confirmed by checking the mRNA expression of Muc-2 which showed a decline (both in ileum and colon) in KO groups fed with the CTR diet and HFD (Supplementary Figure S11). Masson trichrome staining was used to evaluate the degree of tissue fibrosis. Statistical analysis of positive rates in ileum found that the degree of fibrosis was greater in the KO HFD diet group in comparison with WT CTR, WT HFD, and KO CTR groups (Supplementary Figure S6), while there was no significant difference in colon between the groups (Supplementary Figure S7). These results indicated that deletion of *TRIM67* may induce the pathological damage and HFD feeding exacerbates this damage, while deletion of *TRIM67* has no obvious effects on the degree of tissue fibrosis.

### 2.3. TRIM67 Promotes Intestinal Barrier Integrity and Protects from the Worst Effects of HFD

**Figure 3:**
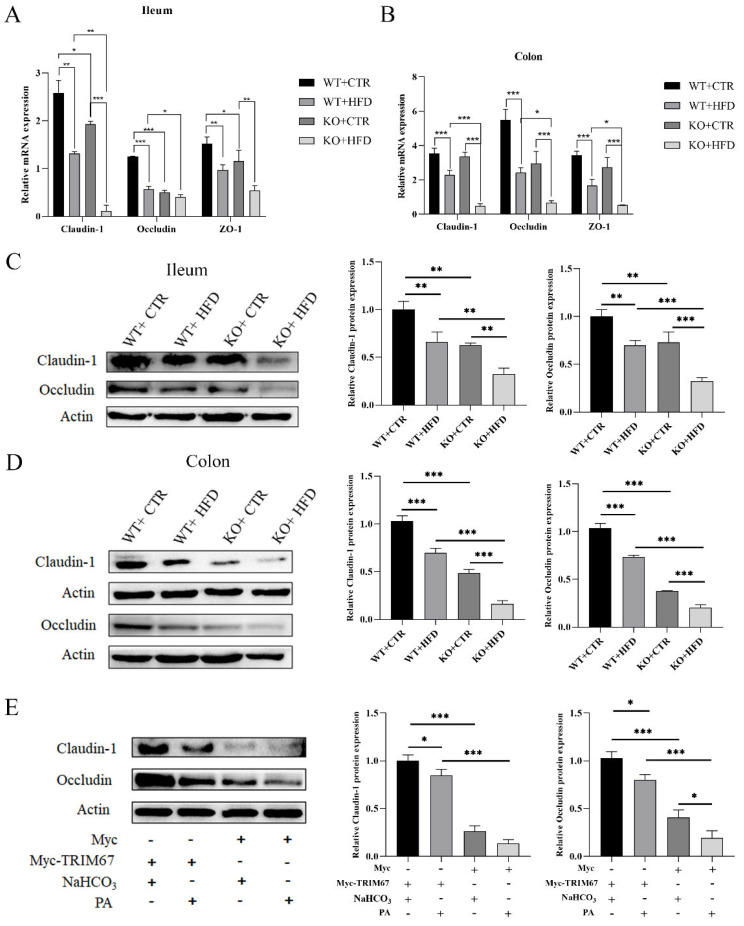
Knockout of *TRIM67* decreases the expression of tight junction proteins and HFD exacerbate the intestinal barrier damage. (**A**) The relative mRNA expression of *claudin-1* (*n* = 5), *occludin* (*n* = 4) and *ZO-1* (*n* = 4) in ileum, and (**B**) The relative mRNA expression of *claudin-1* (*n* = 4), *occludin* (*n* = 5) and *ZO-1* (*n* = 4) in colon. (**C**,**D**) Western blots and quantification of claudin-1 and occludin in ileum and colon (*n* = 3). (**E**) Detection of claudin-1 and occcludin protein and quantification in *Myc-TRIM67* and Myc cell lines supplemented with NaHCO_3_ and PA, “-”: no addition, “+”: addition (*n* = 3). The data are expressed as mean ± SEM. Significance was determined by one-way ANOVA (* *p* < 0.05, ** *p* < 0.01, *** *p* < 0.001).

To explore the further effects of intestinal inflammation and the protective effect of *TRIM67*, we assessed the intestinal permeability by examining the tight junction proteins and endotoxin levels. The expression of epithelial tight junction proteins showed gut permeability [23]. We therefore measured the expression of the major tight junction proteins zonula occludens (ZO)-1, occludin, and claudin-1 in ileum and colon. In ileum, the mRNA expression of *claudin-1*, *occludin,* and *ZO-1* was remarkably lower in the KO CTR diet group than that of the WT CTR diet group, and expression of *claudin-1* and *occludin* was lower in the KO HFD group than in the WT HFD group, while there was no significant difference in the expression of *ZO-1* between the two HFD groups (Figure 3A). Similarly, in colon, the mRNA expression level of *claudin-1*, *occludin*, and *ZO-1* was more down-regulated in the HFD group than in the relevant CTR diet group, and expression was significantly down-regulated in KO HFD group compared to that of WT HFD group; there was no significant difference in expression between the WT CTR diet and the KO CTR diet (Figure 3B). The relative protein expression level in ileum and colon showed that the expression of tight junction proteins (occludin and claudin-1) decreased significantly in HFD fed groups as compared to their relevant CTR diet group, while the KO CTR diet group and KO HFD groups showed lower expression relative to the WT CTR diet and WT HFD groups (Figure 3C,D). These outcomes indicated that KO groups showed decreased expression of tight junction proteins as compared to their relevant WT groups and expression was minimum in HFD feeding groups especially in the KO HFD group. At the same time, we tested the concentrations of LPS in the blood and found that it was highest in the KO HFD group and significantly higher than in the WT HFD group. The concentration in the KO CTR group was also significantly higher than in the control group (Supplementary Figure S12). Similarly, in the in vitro experiments we found that the expression of claudin-1 and occludin was substantially suppressed in Myc groups when compared with *Myc-TRIM67* groups, and a lower expression level was exhibited by PA supplement groups than by NaHCO_3_ groups (Figure 3E), while no such difference was seen in expression of Claudin-1 between the PA and NaHCO_3_ supplement groups of Myc. Taken together, these results elucidated that *TRIM67* promoted the integrity of the intestinal epithelial barrier, and deletion of *TRIM67* increased intestinal permeability which worsened with HFD treatment.

### 2.4. Deletion of TRIM67 can Suppress the Antioxidant Capacity in Ileum and Colon

**Figure 4:**
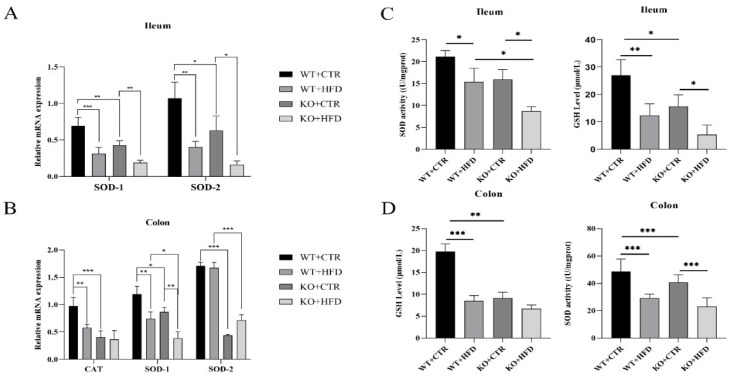
*TRIM67* knockout mice represent suppressed antioxidant capacity in ileum and colon. (**A**) The mRNA expression of *SOD-1* (*n* = 4) and *SOD-2* (*n* = 4) in ileum. (**B**) Relative mRNA expression of *CAT* (*n* = 4), *SOD-1* (*n* = 4) and *SOD-2* (*n* = 5) in colon. (**C**,**D**) SOD activity and GSH level in ileum and colon to check the antioxidant activity (*n* = 8). The data are expressed as mean ± SEM. Significance was determined by one-way ANOVA (* *p* < 0.05, ** *p* < 0.01, *** *p* < 0.001).

To assess the impact of HFD and *TRIM67* on intestinal oxidative stress, we investigated the mRNA expression level of *SOD-1*, *SOD-2* and *CAT* in colon and ileum; superoxide dismutase (SOD) activity and level of glutathione (GSH) were also evaluated. The mRNA expression level of *SOD-1* and *SOD-2* in ileum, and *SOD-1*, *SOD-2*, and *CAT* in colon were down-regulated in KO mice as compared to WT mice when fed with CTR diet. The mRNA expression was lowest in the ileum and colon of KO HFD-fed mice, while there was no significant difference in expression of *SOD-2* and *CAT* in the feed groups of KO mice in colon (Figure 4A,B). Moreover, the SOD activity and GSH level were significantly inhibited in HFD groups relative to those of CTR diet group, while the activity was downregulated in KO CTR diet group as compared to WT CTR group. Decreased expression was obvious in the KO HFD group as compared to other groups. Additionally, in colon the GSH level and SOD activity showed no significant difference between the diet groups of KO mice (Figure 4C,D).

### 2.5. Deletion of TRIM67 Exacerbate Intestinal Inflammation

**Figure 5:**
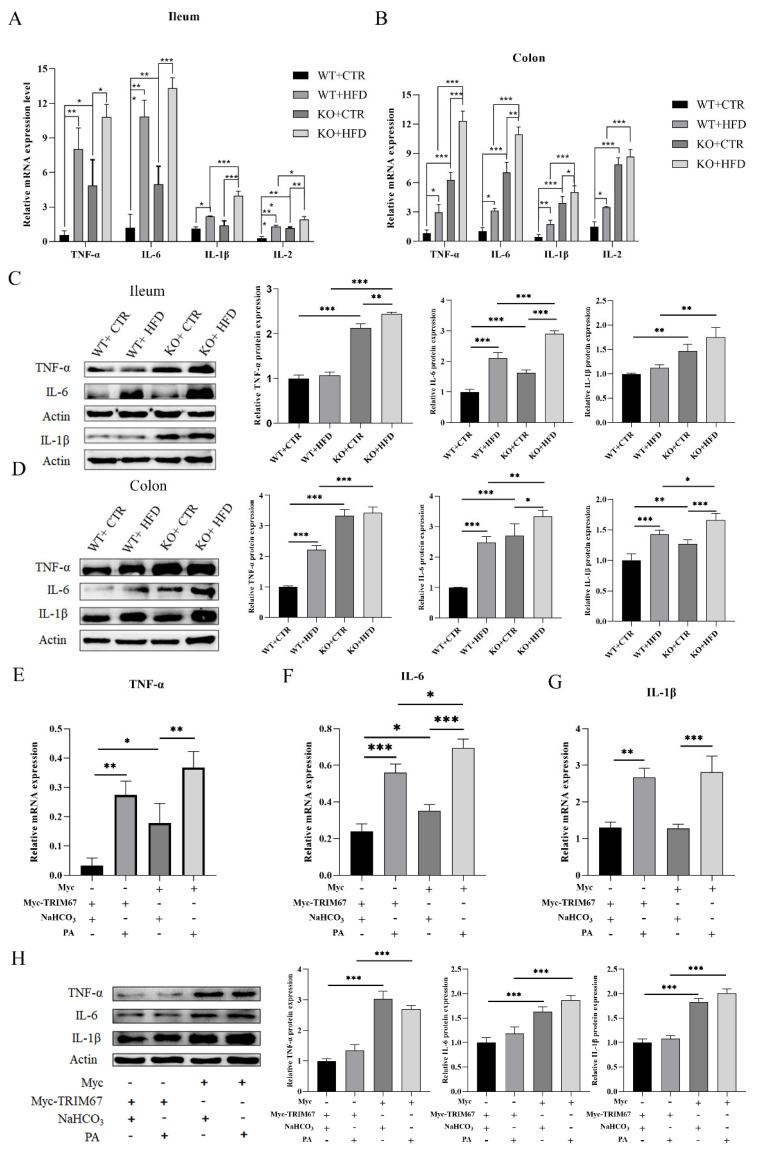
*TRIM67* deletion represents the overexpression of pro-inflammatory cytokines in ileum and colon. (**A**,**B**) Relative mRNA expression of *TNF-α* (*n* = 5), *IL-6* (*n* = 5), *IL-1β* (*n* = 4) and *IL-2* (*n* = 4) in ileum and colon. (**C**,**D**) Western blots and quantification to check protein level of TNF-α, IL-6, and IL-1β in ileum and colon (*n* = 3). (**E**–**G**) Relative mRNA expression of *TNF-α*, *IL-6*, and *IL-1β* in *Myc-TRIM67* and Myc cell lines supplemented with NaHCO_3_ and PA, “-”: no addition, “ + ”: addition (*n* = 4). (**H**) Western blots and quantification display the protein level of TNF-α, IL-6, and IL-1β in Myc and Myc-TRIM67 groups supplemented with NaHCO_3_ and PA, “-”: no addition, “+”: addition (*n* = 3). The data are expressed as mean ± SEM. Significance was determined by one-way ANOVA (* *p* < 0.05, ** *p* < 0.01, *** *p* < 0.001).

To check whether the increased intestinal permeability and oxidative stress was due to intestinal inflammation or not, we examined the inflammatory cytokines in ileum, colon and cell lines. When feeding with HFD, we detected that the inflammatory response was more in the KO group whether fed with CTR diet or HFD. Similarly, when feeding with CTR diet, the inflammatory response was significantly greater in the KO group as compared to the WT group. The KO CTR diet group showed a higher mRNA expression level of inflammatory cytokines (*TNF- α*, *IL-6*, *IL-1β*, and *IL-2*) in ileum (Figure 5A), and colon (Figure 5B) as compared to the WT CTR diet group, while no such change was seen in the expression of *TNF- α* and *IL-6* between the WT HFD and KO HFD groups, and expression of *IL-1β* between the WT CTR and KO CTR groups in ileum. The relative protein expression level of pro-inflammatory cytokines (*TNF- α*, *IL-6*, and *IL-1β*) showed significant increase of inflammatory response in the KO group fed with CTR diet and this response was worsened in KO mice when fed with HFD as compared to all other groups (Figure 3D and Figure 5C). Furthermore, to confirm the protective effect of *TRIM67* in inflammation, we treated the IPEC-J2 with NaHCO_3_ and PA to develop CTR and obesity model, respectively. The expression of selected pro-inflammatory cytokines (*TNF-α*, *IL-6*, and *IL-1β*) was examined by qRT-PCR in cell lines and we observed an up-regulation in expression of pro-inflammatory cytokines in PA stimulated groups greater than that of NaHCO_3_-treated groups, while this expression was considerably higher in the PA treated Myc group as compared to all other groups. On the contrary, the NaHCO_3_-treated Myc group showed significantly higher expression of *TNF-α* and *IL-6* as compared to the NaHCO_3_-treated *Myc-TRIM67* group, while there was no significant difference in the expression of *IL-1β* between the NaHCO_3_-treated Myc group and the NaHCO_3_-treated *Myc-TRIM67* group (Figure 5E–G). We examined the relative protein expression of TNF- α, IL-6, and IL-1β in cell lines: the expression in Myc-TRIM67 with NaHCO_3_ was remarkably lower than in the Myc group with NaHCO_3_ supplements. Moreover, expression of these pro-inflammatory cytokines was substantially more up-regulated in Myc groups than in Myc-TRIM67 groups. However, no significant difference was found in the Myc-TRIM67 group relative to NaHCO_3_ and PA supplements (Figure 5H).

**Figure 6:**
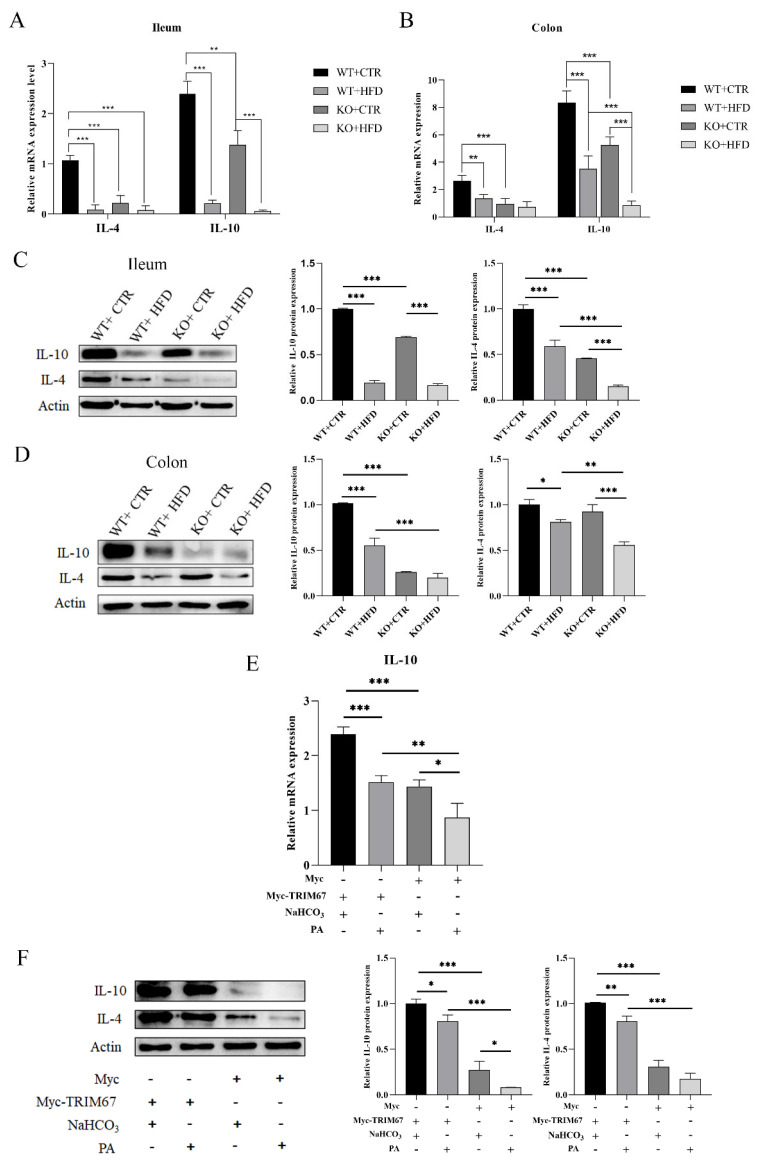
*TRIM67* knockout mice display the lower expression of anti-inflammatory cytokines and HFD feeding exacerbates the condition. (**A**,**B**) Relative mRNA expression of anti-inflammatory cytokines *IL-4* (*n* = 4) and *IL-10* (*n* = 5) in ileum and colon. (**C**,**D**) Western blots and quantification show protein level of IL-4 and IL-10 in ileum and colon (*n* = 3). (**E**) Relative mRNA expression show expression of *IL-10* in *Myc-TRIM67* and Myc group supplemented with NaHCO_3_ and PA, “-”: no addition, “+”: addition (*n* = 4) (**F**) Western blots and quantification of IL-10 and IL-4 proteins in cell lines, “-”: no addition, “+”: addition (*n* = 3). The data are expressed as mean ± SEM. Significance was determined by one-way ANOVA (* *p* < 0.05, ** *p* < 0.01, *** *p* < 0.001).

The mRNA expression of anti-inflammatory cytokines (*IL-10* and *IL-4*) was downregulated in the KO group when fed with CTR diet as compared to the WT CTR diet group, whereas lower expression was associated with the HFD-fed KO group in comparison with the WT HFD group (Figure 6A,B). The WB results revealed a decline in protein expression of IL-10 and IL-4 in ileum and colon of HFD-fed KO mice as compared to all other groups (Figure 6C,D). Similarly, the mRNA expression level of *IL-10* was down-regulated in the NaHCO_3_ supplemented Myc group than in the NaHCO_3_ supplemented *Myc-TRIM67* group. Expression was lowest in the PA supplemented Myc group as compared to all other groups (Figure 6E). Furthermore, when we analyzed the relative protein expression level of IL-4 and IL-10 in cell lines, the most remarkable downregulation was seen in the Myc group with NaHCO_3_ and PA supplements and IL-10 expression was lowest in the Myc group with PA supplement. However, there was no significant difference in expression of IL-4 in the Myc group with NaHCO_3_ and PA supplements (Figure 6F). These findings suggest that deletion of *TRIM67* exacerbates the intestinal inflammation on the CTR diet and worsens the inflammation on the HFD.

## 3. Discussion

In this study we demonstrated that *TRIM67* plays a protective role against inflammation and oxidative stress and maintains the intestinal integrity in ileum and colon of obese mice. Previous studies explain the induction of inflammation, oxidative stress and intestinal barrier dysfunction due to HFD [3,24,25]. In the current study, we demonstrated that *TRIM67* knockout mice showed adverse effects on intestine in HFD-fed mice. We observed that feeding an HFD (starting from the age of 4 weeks) to WT and KO mice for 14 weeks induced pathological damage and increased blood glucose level in both WT and KO mice. Similarly, a decreased number of goblet cells in the KO groups, an increased inflammatory response along with decreasing expression of tight junction proteins ultimately led to lower antioxidant enzymatic activities. The ectopic expression of *TRIM67* in IPEC-J2 cell lines suppressed inflammatory markers and its knockdown in Myc showed higher expression of inflammatory cytokines and tight junction proteins in vitro. The results collectively support the protective role of *TRIM67* in intestinal inflammation and oxidative stress, and in maintaining intestinal integrity.

The HFD effect on the expression of different genes and a previous study about *TRIM69* elucidated that HFD suppressed the expression of *TRIM69* in the hippocampus of mice [26]. In the current study we demonstrated for the first time that the expression of *TRIM67* was downregulated in HFD-fed WT mice as compared to the CTR diet group of WT mice.I It has been noted in previous studies that overexpression of TRIM proteins can increase the membrane repair capacity in muscular dystrophy and restore muscle function and morphology [27,28]. However, it is unclear how HFD suppresses the expression of *TRIM67*. Morphological damage in ileum and colon in the HFD feeding groups can be due to low expression of *TRIM67*. Moreover, our results revealed that the feed intake in KO CTR diet mice was lower than that of WT CTR diet mice, while there was no significant difference in body weight between WT and KO mice fed with control diet. It was further demonstrated that deletion of *TRIM67* had no effect on feed intake or weight gain. Our research found that HFD-fed KO mice showed higher levels of serum total cholesterol, triglycerides, and low-density lipid cholesterol relative to the CTR diet group. However, the high-density lipid cholesterol level was the highest as compared to all other groups, proving the successful development of an obesity model in the HFD fed mice. Collectively these results revealed that deletion of *TRIM67* has no significant effect on feed intake and a HFD suppresses the expression of *TRIM67*.

A HFD affects the intestine and can cause severe pathological effects which are associated with the shortening of intestinal length [29,30]. In our results the length of intestine was lower in the KO CTR diet group as compared to the WT CTR diet group, showing that *TRIM67* knockout affected the intestinal length. Moreover, this study revealed that *TRIM67* knockout mice showed pathological damage in the ileum and colon and a decline in goblet cell count, and that a HFD exacerbated this condition. Previous studies confirmed that a HFD causes intestinal injury [25,31], and an increase in goblet cell count [32]. The current study is in line with these studies, as we found intestinal injury due to a HFD; the effect is worsened in KO mice. However, we observed a contradiction in terms of inhibition of goblet cell count which may be due to deletion of *TRIM67*, diet composition and HFD supplement duration. Further, we observed that the deletion of *TRIM67* has no obvious effects on the degree of fibrosis. The present data indicated that *TRIM67* deletion may induce pathological damage in the ileum and colon and that a HFD exacerbates the intestinal injury in KO mice.

The relationship between diet-induced obesity and its induction of intestinal inflammation is obvious from previous studies [33,34,35]. It was also concluded that TRIM family proteins play a vital role in innate immunity [36,37]. The outcomes of the current study demonstrated that knockout of *TRIM67* increased the expression level of pro-inflammatory cytokines and decreased the expression level of anti-inflammatory cytokines in the ileum and colon, and these results were supported by in vitro experiments by using IPEC-J2 cell lines and Myc with PA supplements. Our study also revealed that KO mice showed an inflammatory response even when fed with the CTR diet and that HFD feeding worsened the inflammatory response. It is not yet clear that how *TRIM67* plays a vital role in ameliorating intestinal inflammation. A previous study identified *TRIM67* as a negative regulator of TNFα-triggered NF-κB activation and showed that downregulation of *TRIM67* may induce the higher expression of inflammatory cytokines [22]. Our data showed that deletion of *TRIM67* induced intestinal inflammation and that the inflammatory response became more severe with HFD feeding.

Tight junction (TJ) proteins signal to the cell interior directly or through recruiting other signaling molecules to regulate cell proliferation, migration, survival and differentiation [38]. Extensive studies have shown that HFD dysregulates tight junction barrier protein expression (ZO-1, claudin, occludin) with or without experimental colitis in ileum and colon tissues of many rodents [39,40,41]. However, the protective effect of *TRIM67* on intestinal integrity was unclear. However, in the current study we showed that deletion of *TRIM67* led to considerably lower expression of TJ proteins in ileum and colon tissues as well as in cell lines. Interestingly, HFD feeding to KO mice as well as PA supplementation to cell lines showed adverse effects on expression of TJ proteins. Our findings revealed that KO mice fed with control diet also exhibited a significant downregulation of TJ proteins. HFD supplementation suppressed the *TRIM67* expression and TJ protein expression became lower in WT HFD-fed mice. The full regulatory mechanism is not yet clear. However, earlier studies have demonstrated that inflammatory cytokines, specially *TNF-α,* contribute to the subsequent inhibition as well as disturbance of TJ proteins [42,43]. Consistent with previous findings, the present study revealed that knockout of *TRIM67* or downregulation of *TRIM67* by HFD feeding promotes the inflammatory response which inhibits the TJ proteins. Therefore, these results indicated that *TRIM67* positively helps maintain the integrity of the intestinal barrier.

Furthermore, it has been found that a HFD induces oxidative stress in intestine by various mechanisms [44,45]. In the current study we showed that deletion of *TRIM67* in mice reduced the antioxidant enzyme activity (GSH and SOD), and that the mRNA expression level of *SOD-1* and *SOD-2* was downregulated on the KO CTR diet as well as with HFD-fed mice. We further revealed that HFD feeding suppressed the expression of *TRIM67.* However, a previous study on humans revealed that DNA methylation of *TRIM67* induces inflammation and oxidative stress [46]. Collectively, we found that deletion of *TRIM67* induces oxidative stress in the ileum and colon, and that HFD feeding exacerbates this condition.

The protective effect of TRIM67 on intestinal tract is not yet clearly understood. This study shows that TRIM67 deficiency leads to intestinal inflammation, and the inflammatory response becomes more serious with HFD feeding. At the same time, this study showed that TRIM67 gene knockout intensified the TJ protein expression inhibition induced by HFD feeding, and that TRIM67 gene up-regulation promoted TJ protein expression. Loss of TRIM67 induces oxidative stress in the gut. Studies have shown that inflammatory cytokines, especially TNF-α, participate in the inhibition and interference of subsequent TJ proteins. Therefore, it is speculated that TRIM67 can regulate tight junction proteins by inhibiting intestinal inflammation, inhibiting the production of inflammatory cytokines, activating signal pathways, and inhibiting intestinal oxidative stress to protect intestinal permeability. How TRIM67 plays its role in protecting intestinal permeability and the specific mechanism by which it affects intestinal inflammation are our next research priority.

## 4. Materials and Methods

### 4.1. Animals

All the animal work was done in agreement with the Animal Care and Use Committee guidelines of Sichuan Agricultural University. All mice used in this study have C57BL/6N strains. Conventional *TRIM67* knockout mice (*TRIM67 -/-*) were generated with CRISPR-Cas9 system targeting on exon 3 and 5 of *TRIM67* as shown in Supplementary Figure S1 (S1) (NCBI Reference sequence: NM_ 330863) by Cyagen Biosciences (Suzhou, China). gRNA target sequence are as follows: gRNA1 (matching reverse strand of gene): TCTGGGTAGGTAACGGCTTCTGG; gRNA2 (matching reverse strand of gene): CAGGCTCAAGGGGGTCTAGACGG: The mice reproduced freely, and the mouse genotype was identified by PCR (WT Forward: 5′-GATGATAGCCATGTAATGCCCACC-3′, Reverse: 5′-TGCCGTTTTCCCCTTCTAAATCAG-3′; KO Forward: 5′-GATGATAGCCATGTAATGCCCACC-3′, Reverse: 5′-CCGTGATATGCTTGCCACAGGTTC-3′). Mice genotype is shown in Supplementary Figure S2 (S2).

### 4.2. Feed

30 healthy, four-week-old wild-type (WT) C57BL/6N male mice were randomly divided into wild type control diet group (WT CTR) fed with control diet, high-fat group (WT HFD) fed with high-fat diet, and thirty healthy. Four-week-old Trim67 knockout (KO) C57BL/6N male mice were randomly divided into an ordinary group fed with control diet (KO CTR) and a high-fat group (KO HFD) fed with a high-fat diet. The mice were fed with the relevant feed of their group for 14 weeks as described in Supplementary Figure S8 (S8). Control diet (CTR) was purchased from Chengdu Dossy Experimental Animals Co., Ltd., and the formula is shown in Supplementary Table S9. The high-fat diet (HFD) was purchased from Medicience Professionals for Lab Animal Diets and the formula is shown in Supplementary Table S10.

### 4.3. Sample Collection

At the end of the 14th week, intraperitoneal injection of 10% chloral hydrate (0.4 mL/100 g) was used for anesthesia, and blood and intestinal tissues of the mice were collected aseptically. A part of each segment of intestinal tissue was fixed in 4% paraformaldehyde, and a part was stored at −80 °C for later use. Blood samples were obtained by cardiac puncture from mice. The collected mouse blood was placed at room temperature for 2 h, centrifuged at 5000 rpm for 10 min at room temperature. The supernatant serum was aspirated and stored in a refrigerator at −80 °C.

### 4.4. Blood Glucose, Blood Lipid and Blood LPS Concentration Detection

After anesthesia, blood glucose level was measured by tail-tip blood method. 0.5~0.8 mL blood was collected from the heart, and the blood container was kept in standing condition at room temperature for 2 h. The supernatant was extracted by centrifugation and stored at −80 °C refrigeration. The collected supernatant was sent to Yibaidao Biological Company (Chengdu, China) for blood lipid detection. Mouse LPS ELISA kit (ML85252-J, Mlbio) was used to detect blood LPS concentration.

### 4.5. Quantitative Real-Time PCR

Total RNA from ileum tissue, colon tissue and cells was extracted according to Animal Total RNA Isolation Kit (RE-03014, Foregene, Chengdu, China) and then 10 μL total RNA was used for reverse transcription, according to RT EasyTM II (With gDNase) (RT-01032, Foregene, Chengdu, China) instructions. Quantitative real-time PCR was conducted by using Real-time PCR Easy TM-SYBR Green I (QP-01014, Foregene, Chengdu, China) and analysis was performed using the Bio-Rad^®^ CFX96 PCR System (Bio-Rad, Hercules, CA, USA) with β-actin or GAPH as internal reference for the relative expression levels of all genes. The primers used in this study for tissues as well as for cells are listed in Table 1 and Table 2.

### 4.6. Western Blotting

Total tissue protein was extracted by mincing the tissues with scissors in tissue lysis buffer (2% SDS with protease inhibitor and phosphatase inhibitor). The protein concentration was measured with BCA protein assay kit (Thermo Scientific Pierce, Waltham, MA, USA). The Western blots processes were carried out as previously described [47]. The SDS-PAGE gel was developed by preparation kit (KGP113, Keygen, Jiangsu, China) instructions. For Western blotting, 5–10 µg protein was loaded into SDS-PAGE gel, followed by protein electrophoresis, membrane transfer, sealing, primary and secondary antibody incubation to obtain protein membrane. It was washed with PBS for three times, and color rendering was done by Super ECL Plus Western blotting solution (GB0001, Bioground, Chongqing, China) for 1–2 min. After that ChampChemi 910 (Sage Creation, Beijing, China) system was used to expose the membrane for image acquisition. The antibodies used for WB are listed in Table 3.

### 4.7. Histopathology

Fresh ileum and colon tissues were fixed with 4% paraformaldehyde (PFA) (fixed at 4 °C for 24 h). The samples were embedded in paraffin and sliced (5 μm). The slices were placed on a glass slide (polylysine coated) followed by dewaxing and rehydration. H&E staining was carried out according to the manufacturer’s instructions (G1120 for H&E, Solarbio, Beijing, China). Glycogen Periodic Acid Schiff (PAS/Hematoxylin) was performed according to manufacturer instructions in Stain Kit (G1281, Solarbio, Beijing, China). The Masson trichrome staining was done by Masson’s Trichrome Stain Kit (G1340, Solarbio, Beijing, China). Finally, the slides were placed in neutral balsam and photographed with a microscope (BX61VS, Olympus, Tokyo, Japan).

### 4.8. Cell Culture

IPEC-J2 cells were obtained from Shanghai Institutes for Biological Sciences, China (SIBS). A 6-well plate dish was taken, and 2 mL (about 1~2 × 10^5^) IPEC-J2 cell culture medium (90% DMEM (A4192101, Gibco, New York, NY, USA), 10% FBS (10099141, Gibco, New York, NY, USA), and 1% Penicillin-Streptomycin Solution (P1400, Solarbio, Beijing, China) were added to each well. The cells were cultured at 37 °C and incubated in 40%~60% CO_2_ density incubator. When the cells’ length reached about 80% they were transfected with two DNA plasmids; one was PRK5-MYC empty plasmid, the other was PRK5-MYC-TRIM67 plasmid. (Transfer solution: solution A: LipoMax TM (32012, SUDGEN, Nanjing, China) taking 4 uL and dissolving in 100 ul opti-MEM (31985070, Gibco); solution B: Plasmids were dissolved in 100 ul opt-MEM; Finally, A and B were mixed). After transient transfection, the efficiency was verified at 48 h to 72 h when the cells were overgrown. In order to simulate cells in control and obese environments, 20 uL of 20 mmol/L NaHCO_3_ (S5240, Solarbio, Beijing, China) and 20 mmol/L palmitic acid (PA) (H8780, Solarbio, Beijing, China) were added into already transfected IPEC-J2 cell culture medium, respectively, and cells were taken out after 24 h stimulation.

### 4.9. Oxidase Activity

After accurately weighing the ileum and colon tissues and adding normal saline, the samples were mechanically homogenated with ice bath and centrifuged to yield the supernatant. 10% homogenate tissues were made according to the ratio of weight (volume = 1:9). Firstly, total tissue protein concentration was determined according to the total Protein Assay Kit (A045-4-2, Nanjing Jiancheng, China). Then the Superoxide Dismutase (SOD) assay Kit (A001-3-2, Nanjing Jiancheng, China) and Reduced Glutathione (GSH) Assay Kit (A006-2-1, Nanjing Jiancheng, China) were used to measure enzyme activity.

### 4.10. Statistical Analysis

The data are presented as mean ± standard error of mean (SEM). Two-tailed Student’s *t* test was performed for all statistical significance analysis using GraphPad prism software. * *p* < 0.05, ** *p* < 0.01, *** *p* < 0.001.

## 5. Conclusions

In conclusion, our data provide important insights into the protective role of *TRIM67* in ileum and colon of obesity induced mice. We demonstrated that *TRIM67* protects from intestinal inflammation, maintains intestinal integrity, and reduces the oxidative stress induced by HFD feeding. Based on the outcomes of the current study, the *TRIM67* can be a valuable therapeutic target. However, further clinical trials are needed to elucidate the mechanisms of *TRIM67* as a protective marker because obesity related metabolic diseases have been increasing in recent years.

## Figures and Tables

**Table 1:** qPCR-related primers sequence.

Gene	Primers (5′-3′)
*β-Actin*	F:AGAGGGAAATCGTGCGTGAC
	R:CAATAGTGATGACCTGGCCGT
*IL-6*	F:CTTCCATCCAGTTGCCTTCTTG
	R:AATTAAGCCTCCGACTTGTGAAG
*TNF-α*	F:ACGGCATGGATCTCAAAGAC
	R:GTGGGTGAGGAGCACGTAG
*TRIM67*	F:GGCGAAGGAGTTTCTGGTTC
	R:TAGCTTCAGGGTGCAGTGATT
*IL-4*	F:CTTCCAAGGTGCTTCGCATA
	R:GATGAATCCAGGCATCGAAA
*IL-10*	F:AAGGGTTACTTGGGTTGCCA
	R:CCTGGGGCATCACTTCTACC
*IL-1β*	F:CCCCAGGGCATGTTAAGGAG
	R:TCTTGGCCGAGGACTAAGGA
*IL-2*	F:CCTGAGCAGGATGGAGAATTACA
	R:TCCAGAACATGCCGCAGAG
*SOD1*	F:AACCAGTTGTGTTGTCAGGAC
	R:CCACCATGTTTCTTAGAGTGAGG
*SOD2*	F:TGGACAAACCTGAGCCCTAAG
	R:CCCAAAGTCACGCTTGATAGC
*CAT*	F:TGGCACACTTTGACAGAGAGC
	R:CCTTTGCCTTGGAGTATCTGG
*Claudin-1*	F:TGGTAATTGGCATCCTGCTG
	R:CAGCCATCCACATCTTCTGC
*Occludin*	F:GTACCCACCAGTGACCAACA
	R:GTTGCTGGAGCTTAGCCTGT
*ZO-1*	F:CGAGGCATCATCCCAAATAAGAAC
	R:TCCAGAAGTCTGCCCGATCAC
	R:GACGCTTATGTTGTTGCTGATGGC

**Table 2:** qPCR primers for CELL.

Gene	Primers (5′-3′)
*GADPH*	F:TCACCAGGGCTGCTTTTA
	R:TTGCCGTGGGTGGAATCATA
*IL-6*	F:TGGGTTCAATCAGGAGACCT
	R:CAGCCTCGACATTTCCCTTA
*TRIM67*	F:CACAAGGCCCAACTGTCTCA
	R:ACCAGAGCATCACACTGAGC
*TNF-α*	F:TCCTCACTCACACCATCAGC
	R:GCCCAGATTCAGCAAAGTCC
*IL-1β*	F:CCAAAGAGGGACATGGAGAA
	R:GGGCTTTTGTTCTGCTTGAG
*IL-10*	F:CTGCCTCCCACTTTCTCTTG
	R:TCAAAGGGGCTCCCTAGTTT

**Table 3:** Antibodies were used in this study.

Name	Company	Country	NO.
Anti-IL-6 Antibody	huabio	China	EM170414 1:1000
IL1β Rabbit pAb	ABclonal	China	A1112 1:1000
Claudin 1 Rabbit Polyclonal Antibody	Beyotime	China	AF6504 1:1000
Anti-Occludin antibody	abcam	Britain	ab216327 1:1000
IL10 antibody	genetex	America	GTX632359 1:5000
Rabbit Anti-IL-4 antibody	Bioss	China	bs-0581R 1:1000
Rabbit Anti-TNF alpha antibody	Bioss	China	bs-2081R 1:1000
TRIM67 Polyclonal Antibody	Proteintech	America	24369-1-AP 1:1000
β-Actin Rabbit mAb	ABclonal	China	AC026 1:100,000

## Data Availability

Source data are provided with this paper and are available from the corresponding author upon reasonable request.

## Supplementary Materials

The following supporting information can be downloaded at https://www.mdpi.com/article/10.3390/ijms23147650/s1.
