# Supplementary material for: Ameliorating Effects of TRIM67 against Intestinal Inflammation and Barrier Dysfunction Induced by High Fat Diet in Obese Mice
Source: Int J Mol Sci. 2022 Jul 11;23(14):7650. doi: 10.3390/ijms23147650 (PMC9317707; doi:10.3390/ijms23147650)
Supplement: Supplementary file 1 [file ijms-23-07650-s001.zip › ijms-1765932-supplementary.pdf]

Supplementary Material

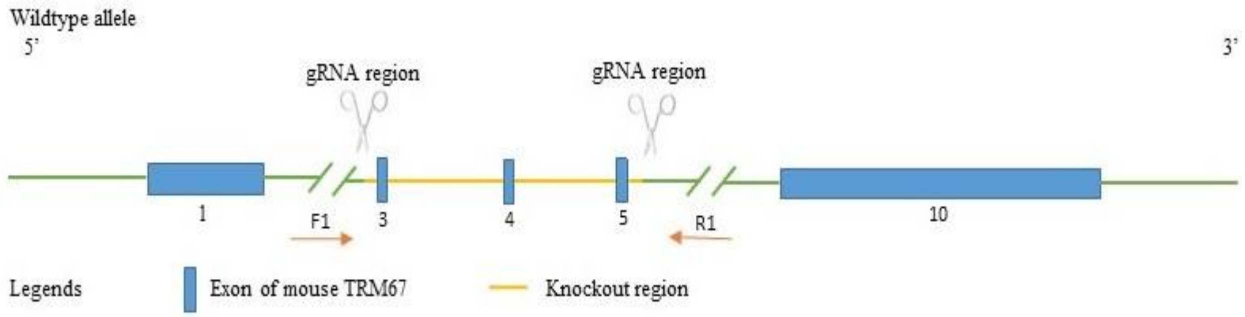

Figure S1: Generation of TRIM67 knockout mice: Targeting exon 3 and 5 of TRIM67 with CRISPR-Cas9 system to generate the TRIM67 knockout mice.

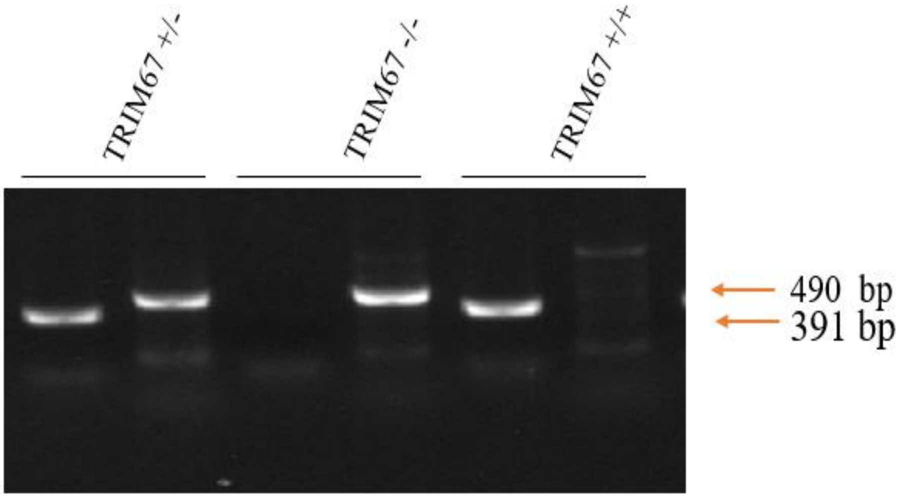

Figure S2: Mice genotype identification: Lanes 1 and 2 are phenotypic TRIM67+/-, lanes 3 and 4 are TRIM67-/-, and lanes 5 and 6 are TRIM67+/+.

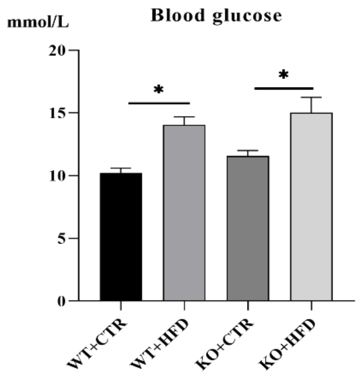

Figure S3: High fat diet increase blood glucose level in WT and KO mice: The blood glucose of mice showed that there is no difference in blood glucose level of WT HFD mice KO HFD mice (n = 8). The data are expressed as mean ± SEM. Significance were determined by one-way ANOVA ($p < 0.05$, * $p < 0.01$, *** $p < 0.001$).

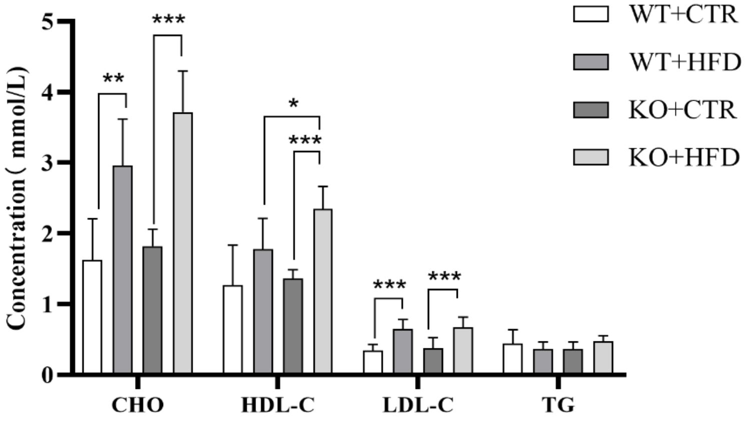

Figure S4: TRIM67 deletion has no obvious effects on blood cholesterol and lipid profile: The blood lipid profile display an increase in the level of cholesterol, high-density lipoprotein (HDL), and low-density lipoprotein (LDL) in HFD groups as compared to their relevant CTR diet group and deletion of TRIM67 has no obvious effects on these parameters, while no significant difference in blood triglyceride level (TG) between the groups ($n = 6$). The data are expressed as mean ± SEM. Significance were determined by one-way ANOVA ($p < 0.05$, * $p < 0.01$, *** $p < 0.001$).

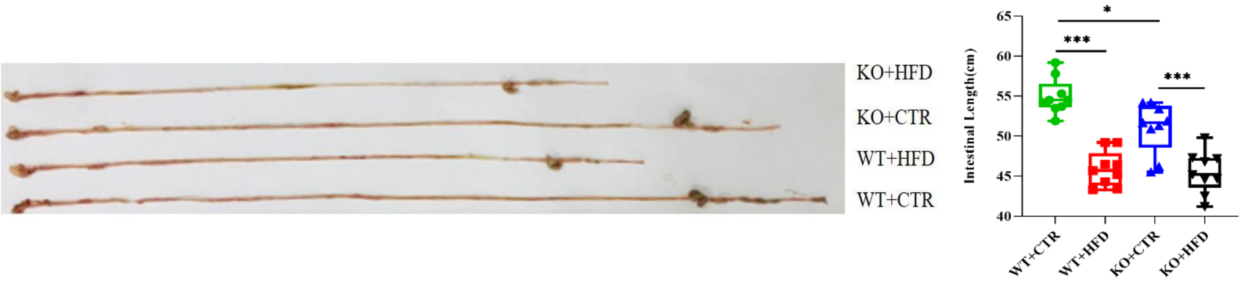

Figure S5: The length of intestine decreases with the deletion of TRIM67 under CTR diet feeding: Representative image and quantification of intestinal length display a decline in intestinal length in KO CTR group as compared to WT CTR group while there was no significant difference between KO HFD and WT HFD groups ($n = 8$). The data were expressed as mean ± SEM. Significance were determined by one-way ANOVA ($p < 0.05$, * $p < 0.01$, *** $p < 0.001$).

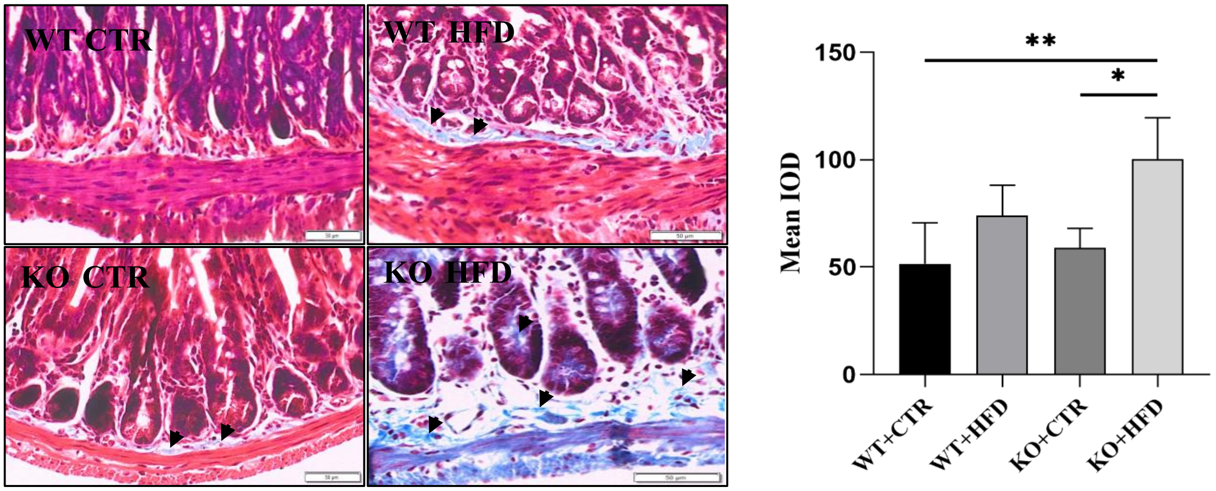

Figure S6: *TRIM67* deletion increase the degree of fibrosis in ileum under HFD feeding: Representative image and statistical analysis of positive rate in ileum show that the degree of fibrosis increased in KO HFD group than that of all other groups (n = 5). The data are expressed as mean ± SEM. Significance were determined by one-way ANOVA (* $p < 0.05$, ** $p < 0.01$, *** $p < 0.001$).

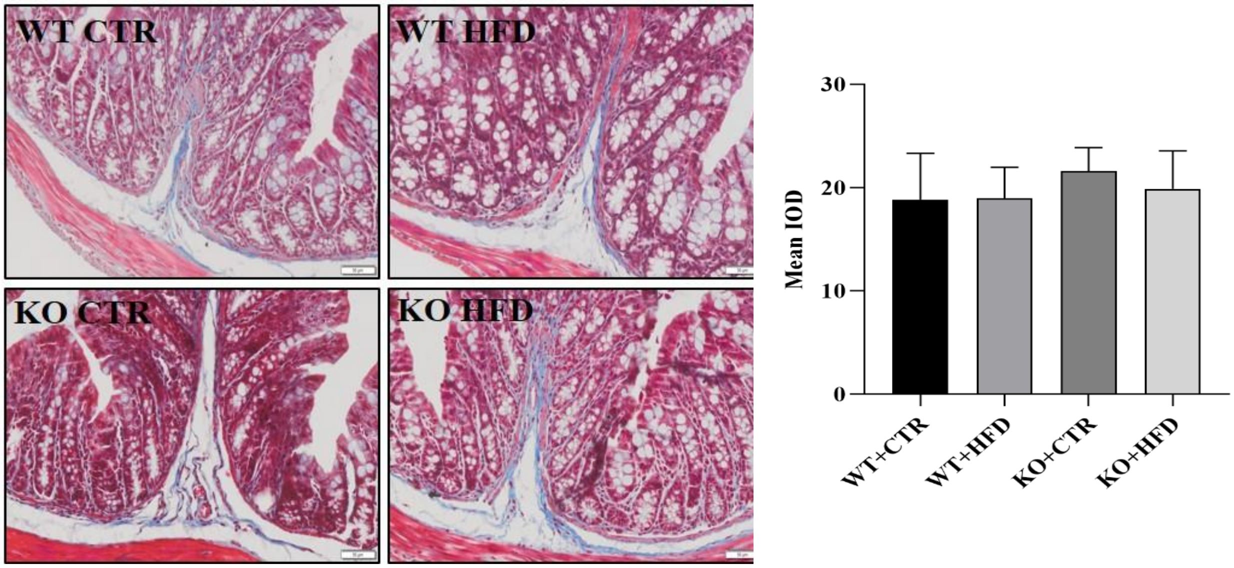

Figure S7: *TRIM67* deletion has no effect on the degree of fibrosis in colon: Representative image and statistical analysis of positive rate in colon show no significant difference between the groups (n = 5). The data are expressed as mean ± SEM. Significance were determined by one-way ANOVA (* $p < 0.05$, ** $p < 0.01$, *** $p < 0.001$).

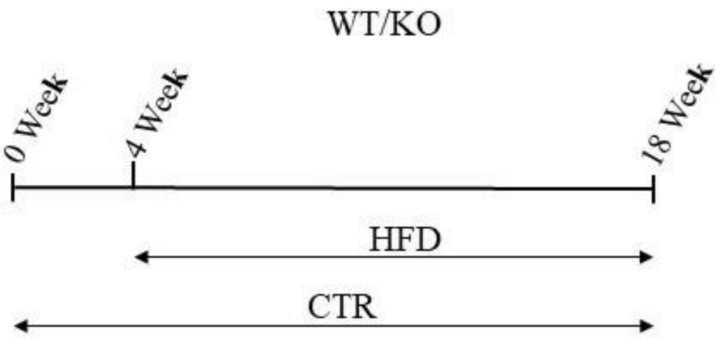

Figure S8: Feed supplementation strategy: Till the age of 4 weeks mice were fed with control diet (CTR), and after 4 weeks the mice of CTR diet group were kept feeding with CTR diet while HFD group mice were fed with HFD after 4 weeks.

Table S9: Control diet (CTR) composition: Control diet nutrients for mice.

Ingredient	Content	Ingredient	Content
Moisture≤	10%	calcium	1.0-1.8%
Crude protein≥	18%	phosphorus	0.6-1.2%
Crude fat ≥	4%	Lysine ≥	0.82%
Crude fiber≤	5%	Methionine+cystine≥	0.53%
Crude ash content ≤	8%	-	-

Table S10: High fat diet (HFD) composition: High fat diet ingredients for mice.

Ingredient	Content	Ingredient	Content
Casein	23.31%	lard	20.68%
L-cystine	0.35%	Complex minerals	3.31%
corn starch	8.48%	Potassium Citrate Monohydrate	1.92%
Maltodextrin	11.65%	multi-vitamins	1.16%
sucrose	20.14%	Choline bitartrate	0.23%
Cellulose	5.83%	pigment	0.005%
Vegetable oil	2.91%	-	-

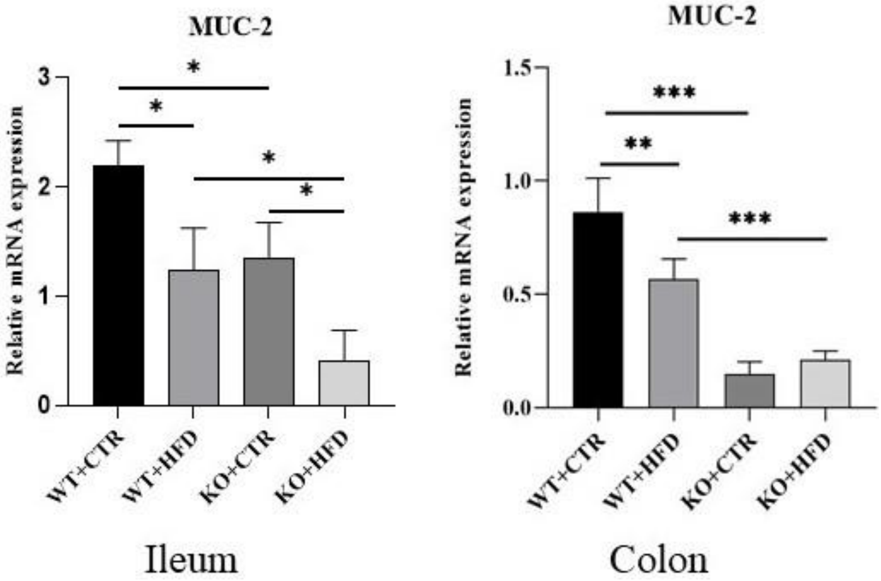

Figure S11: Deletion of *TRIM67* showed a decline in *Muc-2* expression: Relation mRNA expression of *Muc-2* showed that the expression was lowest in KO HFD mice in ileum as compared to all other groups. While expression was lowest in both KO groups in colon as compared to WT groups and there was no significant difference between KO CTR and KO HFD groups in colon (n = 5). The data are expressed as mean ± SEM. Significance were determined by one-way ANOVA (* $p < 0.05$, ** $p < 0.01$, *** $p < 0.001$).

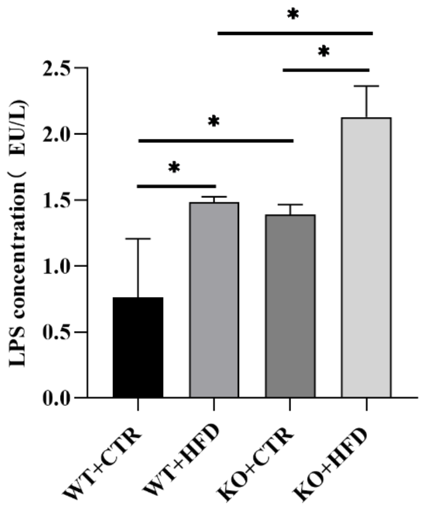

Figure S12: *TRIM67* deletion increase LPS concentration: The concentration of blood LPS in KO HFD group was the highest and significantly higher than WT HFD group. The concentration of KO CTR group was also significantly higher than that of control group (n = 6). The data are expressed as mean ± SEM. Significance were determined by one-way ANOVA (* $p < 0.05$, ** $p < 0.01$, *** $p < 0.001$).
